# Effectiveness of FMT in recurrent *Clostridium difficile* infection

**DOI:** 10.1186/2047-2994-4-S1-P27

**Published:** 2015-06-16

**Authors:** P Grzesiowski, A Hermann, A Dubaniewicz, J Kasprzyk, D Pawlik, Z Zak-Pulawska

**Affiliations:** 1 Preventive Medicine and Rehabilitation Center, Infection Prevention Institute, Warsaw, Poland; 2 Ceynowa Hospital, Wejherowo, Poland; 3 Primary Hospital, Makow Maz., Poland; 4 Primary Hospital, Wolomin, Poland

## Introduction

During last decades C. difficile infection (CDI) has emerged as a most frequent health-care associated cause of morbidity and mortality in developed world. In Europe about 40,000 patients die each year of CDI, in Poland rapid increase of CDI rates has been observed, reaching appr. 11,500 hospitalized cases in 2013.

## Objectives

In mild cases metronidazole is the first-line therapy, with effectiveness of 66%. In severe cases vancomycin is used with cure rate about 78%. Novel therapy with fidaxomicin shows similar clinical cure rates to vancomycin with lower recurrence rates. Alternative method, fecal microbiota transplantation (FMT) for CDI treatment has been reported since 1958 with worldwide response rates of 83-94% as claimed by reviews.

## Methods

We perform the first prospective, non-randomized, multicenter, observational study to estimate the effectiveness of FMT in the treatment of recurrent CDI, including 2 hospitals and one ambulatory center in Poland. The procedure has been proposed for patients with recurrent C. difficile infection that have failed to respond to antimicrobial therapy. All donors, related or unrelated, after obtaining informed consent, have been screened for enteric bacterial pathogens, viruses and parasites according to published guidelines.

## Results

Donor stool after dilution with 0,9% saline has been filtered and resulting suspension has been infused via a nasogastric tube. All patients have signed informed consent according to the written procedure. From 2013 to 2014, in total, 62 patients aged 24-90 years received FMT with resolution of clinical symptoms of 88,7%. Among 55 successfully treated patients, in 76,5% cases recovery has been observed after first FMT procedure, in 14,5% after the second, and in 9% after the third one. There were no statistically significant differences between the cure rates of related and unrelated donor or fresh and frozen samples. There were no severe adverse events observed. Mild diarrhoea, temporary constipation, nausea, flatulence and abdominal pain were most frequently reported side effects.

## Conclusion

Although majority of physicians currently consider FMT as an experimental therapy, we showed that FMT is safe, well tolerated, and highly effective therapy for recurrent CDI.

## Disclosure of interest

None declared.

